# GANDivAWeb: A web server for detecting early folding units ("foldons") from protein 3D structures

**DOI:** 10.1186/1472-6807-8-15

**Published:** 2008-03-07

**Authors:** Thomas Laborde, Masaru Tomita, Arun Krishnan

**Affiliations:** 1 ENSIERB, Bordeaux, France; 2 Institute for Advanced Biosciences, Keio University, 14-1, Baba-Cho, Tsuruoka, Yamagata-ken, 997-0035, Japan

## Abstract

**Background:**

It has long been known that small regions of proteins tend to fold independently and are then stabilized by interactions between these distinct subunits or modules. Such units, also known as autonomous folding units (AFUs) or"foldons" play a key role in protein folding. A knowledge of such early folding units has diverse applications in protein engineering as well as in developing an understanding of the protein folding process. Such AFUs can also be used as model systems in order to study the structural organization of proteins.

**Results:**

In an earlier work, we had utilized a global network partitioning algorithm to identify modules in proteins. We had shown that these modules correlate well with AFUs. In this work, we have developed a webserver, GANDivAWeb, to identify early folding units or "foldons" in networks using the algorithm described earlier. The website has three functionalities: (a) It is able to display information on the modularity of a database of 1420 proteins used in the original work, (b) It can take as input an uploaded PDB file, identify the modules using the GANDivA algorithm and email the results back to the user and (c) It can take as input an uploaded PDB file and a results file (obtained from functionality (b)) and display the results using the embedded viewer. The results include the module decomposition of the protein, plots of cartoon representations of the protein colored by module identity and connectivity as well as contour plots of the hydrophobicity and relative accessible surface area (RASA) distributions.

**Conclusion:**

We believe that the GANDivAWeb server, will be a useful tool for scientists interested in the phenomena of protein folding as well as in protein engineering. Our tool not only provides a knowledge of the AFUs through a natural graph partitioning approach but is also able to identify residues that are critical during folding. It is our intention to use this tool to study the topological determinants of protein folding by analyzing the topological changes in proteins over the unfolding/folding pathways.

## Background

It is recognized that small regions of proteins tend to fold independently and are then stabilized by interactions between these distinct subunits or modules. The dissection of proteins into structurally independent and functionally distinct subunits led to the idea that proteins can be considered as collections of smaller units such as domains [1]. Different definitions of domains have been in existence [2]. While some define a domain as a recognizable substructure within a protein and connected to other domains by very few structural elements such as a loop or a helix, others define domains as a parts of a protein molecule that behave in a quasi-independent manner and are considered as cooperative units in protein folding [3,4]. A further definition describes a domain as a relatively compact part of a protein that is characterized by its own pattern of intramolecular collective dynamics and which are distinguishable from those of other domains [5-8]. In the belief that an unequivocal definition of a module must be based on the most fundamental property of protein 3D structure, namely, the adjacency matrix of inter-residues contact, we adopted a network representation of the protein.

In an earlier work [9], we had used a well-established, global method for identifying modules in networks [10]. The algorithm converges towards the maximization of the modularity of the given protein network; the network being defined by an adjacency matrix with a 1 denoting the existence of residue-residue contacts and a 0 for non-contacts. Maximizing the modularity score, as defined by Guimera *et al*. [10] results in maximizing intra-module contacts while minimizing the inter-module contacts. The modularity *M *is given by

$$M=\sum_{s=1}^{N_{m}}\frac{l_{s}}{L}-{\left(\frac{d_{s}}{2L}\right)}^{2}$$

where *N*_*m *_is the number of modules, *L *is the number of links in the network, *l *is the number of links between nodes in module *s *and *d *is the sum of the degrees of the node in module *s*. In doing so, this allows the representation of the residues of the protein in terms of their intra-module degree, *z *and participation coefficient, *P*., which are given by

$$\begin{array}{l} z_{i}=\frac{\kappa_{i}-\kappa_{s_{j}}^{-}}{\sigma_{\kappa_{s_{j}}}}\\ P_{i}=1-\sum_{s=1}^{N_{M}}{\left(\frac{\kappa_{is}}{k_{i}}\right)}^{2} \end{array}$$

where *κ*_*i *_is the number of links of residue *i *to other residues in its module *s*_*i*_, ${\bar{\kappa }}_{s_{j}}$ is the average of *κ *over all residues in module *s*_*j*_, $\omega_{\kappa_{s_{j}}}$ is the standard deviation of *κ *in module *s*_*j*_, *κ*_*is *_is the number of links of node *i *to nodes in module *s *and *k*_*i *_is the total degree of node *i*.

We demonstrated that the labeling of residues in terms of these invariants, allowed for information rich representations of the studied proteins as well as to sketch a new way to link sequence, structure and the dynamical properties of proteins. We discovered a strong invariant character of protein molecules in terms of *P*/*z *characterization, pointing to a common topological design of all protein structures. This invariant representation, applied to different protein systems enabled us to identify the possible functional role of high *P*/*z *residues during the folding process. Effectively, this invariance is a cartographic representation of the contact network for proteins and is represented by the plot of the residues in the *P *- *z *space. Since it is identical for all the proteins, it does not embed any structural peculiarities or information for separating between different protein folds [11,12].

We also observed that the modules identified using the procedure outlined above correlated well with early folding units or "foldons" and thus a knowledge of the modules existing in a given protein can help to identify residues that are critical for folding.

A significant use for the modules identified using our methodology is for the development of algorithms for protein 3D structure determination. In addition knowing the modules for a protein can help in the understanding of the folding pathway for that protein since residues with high |*P*/*z*| values tend to be protected during transition state and hence are fixed early in the folding process.

The modules can also be used for engineering new enzymes which is typically carried out by building a chimera of multiple proteins by cutting and pasting sequences from the respective proteins. A knowledge of the modules can guide the cuts in order to obtain chimeras that can fold in-vitro. In addition models of such early folding units can be invaluable in understanding the biochemical pathways of diseases that are known to be pathological through partially folded forms of proteins leading to the development of therapeutics.

## Implementation and Results

### GANDivAWeb

**Figure 1:**
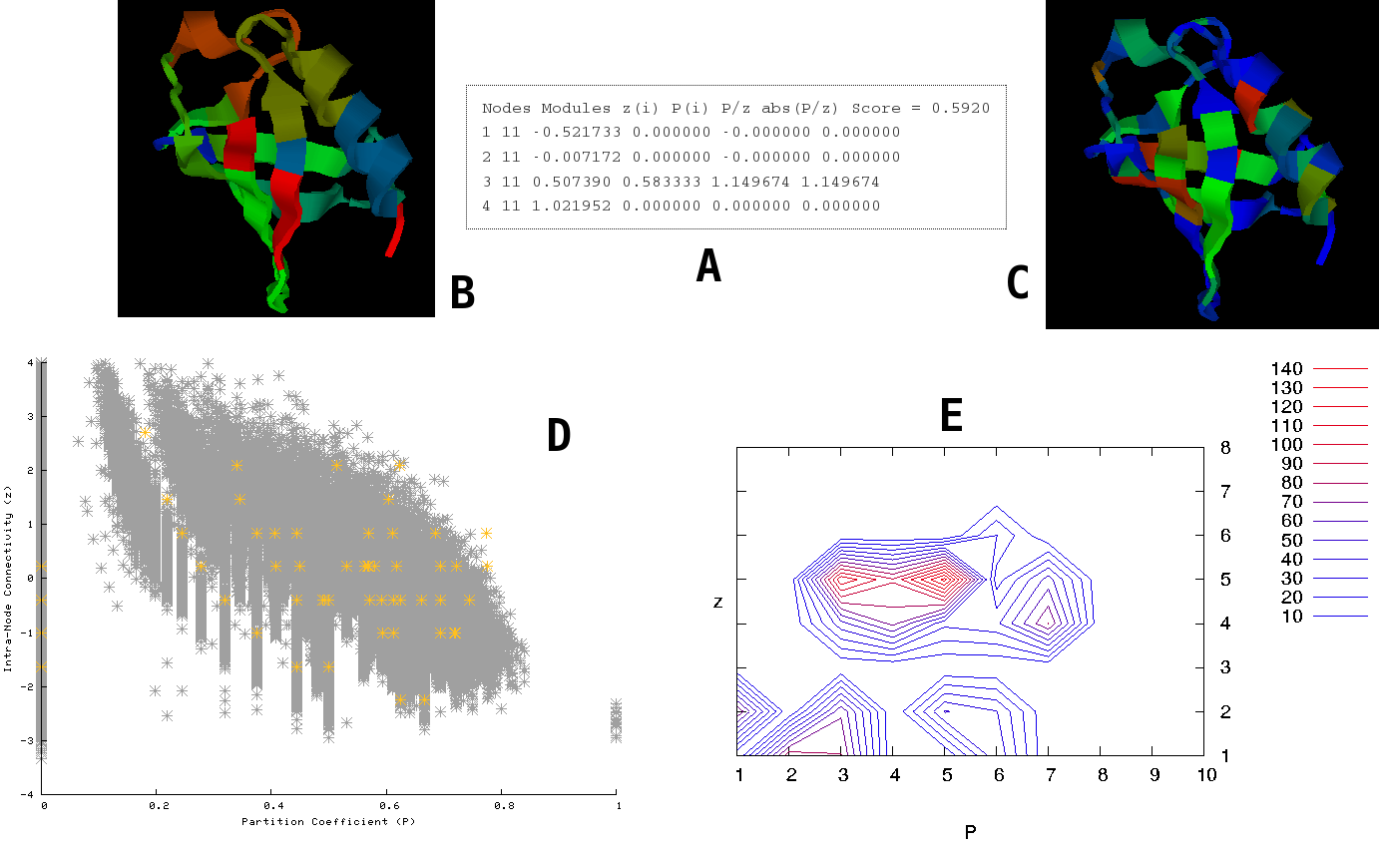
**The figure shows the results emailed back to the user. **(A): The results file, (B): Cartoon representation of the protein with the residues colored according to the modules, (C): Cartoon representation of the protein with the residues colored according to the |*P*/*z*| value, (D): Scatter plot of the residues on the *P *- *z *plane, (E): Contour plots of the distribution of the buried and surface residues (Relative Accessible Surface Area (RASA) plot). See text for detailed explanations of the different figures.

The GANDivAWeb webserver is based on the GANDivA (Genetic Algorithm-based Network modularity DetectIVe Algorithm) algorithm which is an implementation of the Guimera algorithm [10] that uses a genetic algorithm (GA) to optimize the modularity score, instead of simulated annealing which was used in the original algorithm. The algorithm has been written in C and was developed using the parallel genetic algorithm PGAPACK [13], the GNU Scientific Library [14] and the OpenMPI [15] and MPICH [16] MPI [17] implementations. The webserver has three main functionalities:

1. It displays the results from the application of the modularity algorithm GANDivA on a set of 1420 single-chain, globular proteins that were used as the basis for the work done in [9].

2. The main functionality of the website however, is the ability to process an uploaded protein structure file (in PDB format) using GANDivA and to send the results back to the user. Since GANDivA is a stochastic algorithm, the algorithm is run a number of times and the best results, as denoted by the largest modularity score obtained, is emailed back to the user. The user can set certain parameters like the maximum number of modules to be determined, the number of times to run the algorithm, the number of generations for the genetic algorithm as well as the number of generations for the fitness score to remain constant before it is assumed to have converged to a solution. An algorithm that takes into account the size of the protein (number of amino acid residues) as well as the number of jobs in the queue for the cluster, notifies the user about the expected time required for the completion of the job. The results emailed back to the user are made up of the following parts:

• A results file that contains the details of the modular decomposition of the protein. The results file 1(A) contains the modularity score in the first line. This is succeeded by the following columns: residue number, module number, intra-module connectivity (*z*), inter-module participation coefficient (*P*), *P*/*z*, |*P*/*z*|. The second column indicates the module to which each residue belongs. In addition to the results file, five different figures are generated.

• Along with the results file four figures are also included in the final results. The first two figures (Figures 1(B) and 1(C)) show cartoon representations of the protein with the residues colored according to the modules that they belong to and the |*P*/*z*| value, respectively. High |*P*/*z*| valued residues act as structural stabilizers and have been found to be correlated with the residues that are protected in the transition phase.

• The third figure (Figure 1(D) shows a scatter plot of the residues on the *P *- *z *plane. This scatter plot is overlaid on the scatter plot of the residues for the 1420 proteins studied in [9]. Marked deviation from the typical "dentist's chair" shape for a particular protein suggests non-native contacts.

• The fourth (Figure 1(E) and fifth (figure not shown) figures show contour plots of the distribution of the buried and surface residues (Relative Accessible Surface Area (RASA) plot) and the hydrophobic and polar residues over the topological *P *- *z *space, respectively. The distributions were obtained by first calculating the residue accessible surface area (RASA) and hydrophobicities respectively for each residue followed by partitioning the *P *- *z *space into an 8 × 10 grid and then calculating the mean value of the RASA and hydrophobicities in each bin. The mean value for each cell of the 8 × 10 grid was then plotted as a contour plot. Significant differences have been observed between these distributions for native and decoy proteins, with native distributions showing more structure (inclusive of two clearly defined regions where hydrophobic/low RASA residues are embedded).

3. The third functionality of the webserver is in displaying the results obtained using GANDivA. The user can upload the PDB file and the results file (obtained from functionality 2 above) and view the results in the embedded JMol viewer. For both functionalities 1 and 3, the user can choose to view the protein colored by the modules that the residues belong to or by the |*P*/*z*| values. The user can also choose to view only hydrophobic, only polar or all the residues. Additionally, the viewer can choose the graphical representations of the protein that are a part of the embedded viewer.

**Figure 2:**
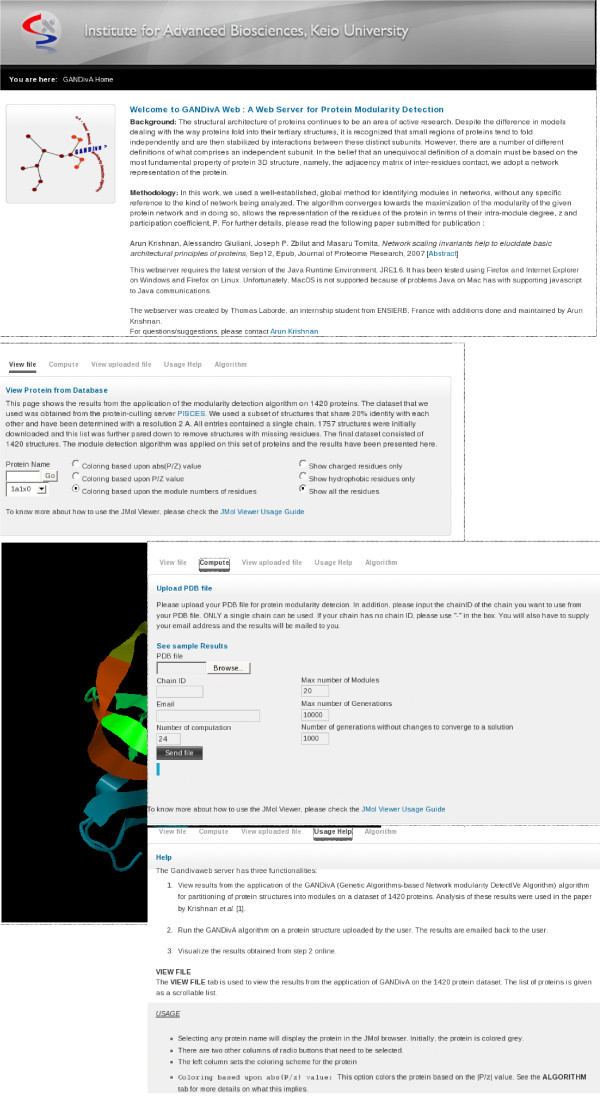
The GANDivAWeb User Interface: The figure shows the user interface for gandivaweb with snapshots of the different tabs corresponding to the main functionalities shown.

Figure 2 shows screenshots of the different tabs pertaining to the various functionalities of the GANDivAWeb server.

## Discussion

The GANDivAWeb server has been designed to partition any given PDB structure into modules. The modules thus obtained correlate well with autonomous folding units. Additionally, high |*P*/*z*| valued residues are identified. These residues have been shown [9] to correlate well with residues that are protected early during the folding process. A knowledge of such units can help in understanding the folding process. It can also be used for engineering enzymes as mentioned earlier. Enzymes are typically engineered by cutting and pasting sequences from multiple proteins. Knowing the "natural" boundaries of protein folding modules can guide the "cuts" required in order to engineer proteins that can fold in-vitro. Moreover, the knowledge of early folding units can help in understanding the biophysical and biochemical causes of diseases that are caused by the misfolding of proteins.

## Conclusion

We believe that the GANDivAWeb server will be of immense use to scientists interested in the phenomena of protein folding and those studying the architectural and structural organization of proteins.

Additionally, the site will also be useful for scientists interested in the engineering of novel enzymes by providing them with a modularized view of the protein.

## Availability

The webserver can be found at 

## Authors' contributions

AK wrote the main algorithm and wrote the paper. TL designed and implemented the webserver and also helped in the writing of the paper. MT was in charge of the overall project.
